# Designing a potent multivalent epitope vaccine candidate against *Orientia tsutsugamushi* via reverse vaccinology technique - bioinformatics and immunoinformatic approach

**DOI:** 10.3389/fimmu.2025.1513245

**Published:** 2025-02-13

**Authors:** Subhasmita Panda, Subrat Kumar Swain, Basanta Pravas Sahu, Soumya Ranjan Mahapatra, Jyotirmayee Dey, Rachita Sarangi, Anu Vinod Ranade, Namrata Mishra

**Affiliations:** ^1^ Institute of Medical Sciences and SUM Hospital, Siksha O Anusandhan University, Bhubaneswar, India; ^2^ School of Biological Sciences, The University of Hong Kong, Hong Kong, Hong Kong SAR, China; ^3^ School of Biotechnology, Kalinga Institute of Information and Technology (KIIT) University, Bhubaneswar, India; ^4^ Department of Basic Medical Sciences, College of Medicine, University of Sharjah, Sharjah, United Arab Emirates

**Keywords:** multiplex vaccinology, scrub typhus, protein-protein docking, immune simulation, in-silico cloning, molecular dynamic simulation

## Abstract

Scrub typhus is a life-threatening, undifferentiated febrile illness caused by a gram-negative bacterium, *Orientia tsutsugamushi*. The bacterial strain is a global health concern that should be considered. Despite several years of effort for the development of an effective immunogenic vaccine, no successful licensed vaccine is available. The aim of the study is to construct an epitope response using a reverse vaccinology approach. The TSA56 and ScaA proteins combined can be the most promising subunit vaccine candidates against *O. tsutsugamushi*. B-cell, CTL, and HTL epitopes were predicted, and subsequently, all the epitopes were linked by KK, AAY, and GPGPG linkers, respectively, along with an adjuvant at the N-terminal region. Furthermore, molecular docking and MD simulations were performed that exhibited a higher affinity towards TLR-2. A total of 16 linear B-cells, 6 CTL, and 2 HTL epitopes were identified and validated. The final vaccine construct showed high antigenicity, stability, and solubility. Molecular docking and MD simulations indicated strong binding interactions with TLR-2 and a stable vaccine-receptor complex. The expression of the vaccine in pET28a (+) vector was successfully implemented via in silico cloning as well as significant results from immune simulation demonstrated the efficacy of the vaccine in the immune cell interaction during the innate and adaptive immune responses immune simulation. In conclusion, the outcome suggested that the newly developed vaccine will be a promising candidate for controlling and providing definitive preventive measures against scrub typhus if further investigation is conducted experimentally.

## 1 Introduction

Scrub typhus, a neglected tropical disease, is caused by the gram-negative bacteria *Orientia tsutsugamushi* (*O. tsutsugamushi*), rising rapidly in various endemic countries and becoming a serious health concern (1, 2). Despite being recognized as early as 313 A.D. and causing a significant threat to billions of people across various regions of Asia and Australia, this disease remains underdiagnosed and underreported. Historically, it was confined to the “Tsutsugamushi triangle’, an area covering 13 million km^2^ that extends from Russia to Japan in the north, Northern Australia in the south, and Pakistan and Afghanistan in the west (3). However, confirmed cases have now emerged beyond the traditional countries, including Dubai, Chile, part of Africa, and Peru (4). The diversity in species and epidemic characteristics across other countries like China, Japan, Taiwan, Thailand, Hong Kong, and South Korea (1). Between January 2010 and December 2019, Taiwan reported 4,374 confirmed cases of scrub typhus, consisting of 4,352 domestic cases and 22 imported cases. Analysis of the epidemiological features revealed a significant male predominance (2,699 males vs. 1,675 females) (5). A study reported 6,338 reported ST cases, of which 304 were laboratory-confirmed. Incidence rates rose significantly over the years, from 0.03 per 100,000 in 2006 to 1.12 per 100,000 in 2021, with the highest rates occurring among farmers (6).

Vaccines have evolved over the years, directing from those that use the entire organism either killed or live-attenuated to those that are based on the smaller segments of the organism like toxins, purified antigens, subunits, and synthetic peptides. As genome sequencing became increasingly prevalent, it ushered in a new era in vaccinology known as reverse vaccinology (7). Multi-epitope subunit vaccines have been developed over the past few decades using reverse vaccinology techniques to combat a wide variety of infections such as *Acinetobacter baumannii* (8), *Pseudomonas aeruginosa* (9), *Klebsiella pneumonia* (10), *Helicobacter pylori* (11), *Mycobacterium tuberculosis* (12) and many more. These vaccines are effective in both *in vitro* and *in vivo* mouse model, making them viable alternatives to the cost and time-consuming process of developing vaccination in the trial-and-error process of the conventional way.

In the past few decades, demand for the use of active immunotherapy has increased, such as an epitope-based vaccination, for the treatment of different diseases (13). Prevailing epitope vaccination deficits can be mitigated by a variety of approaches including enhancing the number of antigenic epitopes that can be targeted by an immunogenic adjuvant or a carrier protein (14, 15).

Five autotransfer domain-containing proteins (ScaA-ScaE) and immunogenic surface antigen proteins such as TSA22, TSA47, and TSA56 are encoded by the 2.1Mbp genome of the gram-negative bacterium *O. tsutsugamushi*. ScaA facilitates bacterial adherence to the host cell and exhibits a high level of strain-specific conservation in its passenger domain (16). Patients develop strain-specific antibodies due to four variable domains of TSA56. Thus, potential vaccine candidates, including TSA47, TSA56, and ScaA, have been investigated (17, 18). Toll-like receptors are the most extensively researched Pathogen Recognition Receptors (PRRs); they are accountable for recognizing pathogen-associated molecular patterns (PAMPs). TLR2 is frequently used as a target for adjuvants because its activation can enhance both innate and adaptive immune responses. TLR2 activation can promote a strong antigen-presenting cell response and subsequent T-cell activation. In this research, the immunoinformatics and reverse vaccinology approach was applied to develop a potential multiepitope chimeric vaccine against the *O. tsutsugamushi* bacterium to prevent the spread of scrub typhus.

## 2 Materials and method

### 2.1 Sequence availability and proteome retrieval

Highly virulent TSA56 (accession no. SPR11258.1) and ScaA (accession no. SPR07654.1) proteins from all the available complete genome sequences of *O. tsutsugamushi* were retrieved from the NCBI database, among which the highly antigenic strain Gilliam was selected.

### 2.2 Screening of potent epitopes

B cell epitopes play a key role in generating long-term humoral immune response and memory cells by activating B lymphocytes. Linear B cell epitopes were predicted by using ABCPred (19) with a 10-mer window length. The NetMHCpan 4.1 server (20) was used to predict the MHC-I restricted CD8+ cytotoxic T-lymphocyte (CTL) epitopes of the chosen protein sequence. CTL epitope prediction was studied across 12 MHC class-I supertypes (HLA-A01:01, HLA-A02:01, HLA-A03:01, HLA-A24:02, HLA-A26:01, HLA-B07:02, HLA-B08:01, HLA-B27:05, HLA-B39:01, HLA-B40:01, HLA-B58:01, HLA-B15:01). In response to invading pathogens, helper T lymphocytes can activate either humoral or cellular immune responses. The IEDB server was used to predict HTL epitopes with human HLA cells as the default parameter. Scores below 50 nM for the IC50 indicate a strong binding affinity.

### 2.3 Evaluation of epitopes for vaccine construct and assessment

Antigen, non-allergen, non-toxic, and immunogenicity were all taken into account while selecting the epitopes by using VaxiJen v2.0 (21), AllerTop v2.0 (22), ToxinPred (23) and IEDB servers respectively. Cholera toxin subunit B (ACO36766.1), a potential adjuvant, was linked to the N-terminus of the construct with the help of EAAAK peptide linker to accelerate the immune response, followed by selected LBL, CTL, and HTL epitopes with appropriate linkers KK, AAY, and GPGPG to aid the amino acids in folding into appropriate conformations with maximum flexibility. The solubility of the vaccine design was calculated using the SolPro service (24). ExPASyProtParam server (25) was then used to evaluate the developed vaccine construct for its physicochemical characterization.

### 2.4 mRNA

With the help of the Mfold web server, the multi-epitope vaccine RNA secondary structure was anticipated (26). With minimal ΔG thermodynamics, this server provides true positive bps prediction.

### 2.5 Cluster analysis of MHC alleles

Similar binding specificities among MHC-I and MHC-II molecule alleles can be determined by cluster analysis which was done by using MHCcluster 2.0, a web-based server (27). All of the HLA supertype representatives were chosen during the analysis, and the number of peptides to be included was kept constant at 50,000.

### 2.6 Conservancy analysis and global population coverage

Conservancy analysis was performed on the anticipated epitope using the IEDB analysis resource (28) to predict the degree of similarity within the serotypes of *O. tsutsugamushi* at a sequence identity threshold of 60%. Due to polymorphism, MHC molecules may demonstrate substantial variations among populations. The IEDB population coverage server was used to determine whether or not the chosen CTL and HTL epitope alleles were represented in the general population (29).

### 2.7 Homology modelling, assessment and validation of vaccine construct

The PSIPRED 4.0 web server was employed to provide accurate predictions about the secondary structure of the vaccine construct (30). The tertiary structure of the vaccine sequence was obtained using the Robetta server (31). PyMol was used to display the 3D structure of the vaccine construct. GalaxyRefine2 (32) server was used to improve the final structure. The development of the Ramachandran plot using PROCHECK (33) and SWISS_MODEL (34) was used to check the model quality. The Ramachandran plot is used in this server to foresee the probability that amino acids form a secondary structure and to illustrate the quality of models by the proportion of amino acids in the favored, allowed preferred, and outlier ranges. The validation was additionally approved by the SAVES server using ERRAT (35) and Verify3D (36).

### 2.8 Discontinuous B-cell epitope prediction

Over 90% of B cell epitopes were found to be discontinuous. The confirmed three-dimensional structure of conformational B-cell epitopes is predicted by the web-based server Ellipro (37). Compared to other structure-based epitope prediction techniques, Ellipro came out on top, with an AUC value of 0.732 as the best computation for any protein.

### 2.9 Disulfide bond engineering

The DbD2 web server (38) was used to create a reasonable disulfide bond in the protein structure and assess whether they were consistent in terms of proximity and geometry. As the proteins are very dynamic, mutations can affect the structure and consequently the function of the protein.

### 2.10 Molecular docking

To predict the interaction and binding affinity between the designed vaccine and human Toll-like receptor-2 (TLR-2), molecular docking is an especially feasible and basic technique. To facilitate molecular docking, the crystal structure of TLR-2 (PDB id: 3a7c) was obtained from the protein data bank. The molecular docking was performed with ClusPro 2.0 employing novel FFT correlation, grouping the best energy conformations, and analyzing cluster stability with a short Monte Carlo simulation to predict the interaction between two proteins (39). The best-docked conformations of the proteins and peptides were visualized using the PyMol visualization tool.

### 2.11 Molecular dynamic simulation of the docked complex

Molecular dynamic (MD) simulation was used in the present study to better understand the protein-protein docking complex that performed most effectively. The MD simulation was carried out using GROMACS 2019 package (40) and OPLS-e force field, with the TIP3P water model used to solvate the protein complex. Sodium and chloride ions were introduced to get a neutral physiological salt content, and the system energy was minimized using the steepest descent technique. To keep the lengths of the covalent bonds constant during the simulation, the Linear Constraint Solver (LINCS) method was utilized. For the long-range electrostatic interactions, the Particle Mesh Ewald (PME) method was used, and for the short-range coulomb and Van der Waals interaction, we settled on a radius of 0.9 nm. After that, equilibrations were performed for both systems at 100 ps NVT [constant number of particles (N), volume (V), and temperature (T)] and 100 ps NPT [constant number of particles (N), pressure (P), and temperature (T)]. After running 50 ns of simulations using PBC, we analyzed the results with GROMACS modules and used xmgrace to create the relevant charts and figures.

### 2.12 Codon optimization and in silico cloning

To enable the production of the vaccine construct in a chosen expression vector, reverse translation and codon optimization was carried out using the Java Codon Adaptation Tool (JCat) website (41). Since the final vaccine design derives its sequence from human DNA, it required some codon optimization so that it could be expressed in *E. coli* strain K12 host. Codon adaptation index (CAI) and GC content (%) are two measures of protein expression that are provided in JCat output. Adding HindIII and BamHI restriction sites to the N and C-terminus of the DNA sequences of the projected vaccines allowed for their cloning in the *E. coli* pET-28a (+) vector, yielding the final vaccine constructs with the optimal gene sequence. The final step in ensuring the expression of the vaccine was to use the SnapGene tool to insert the optimized DNA sequences with restriction sites into the pET-28a(+) vector.

### 2.13 Immune simulation of vaccine

**Figure 1:**
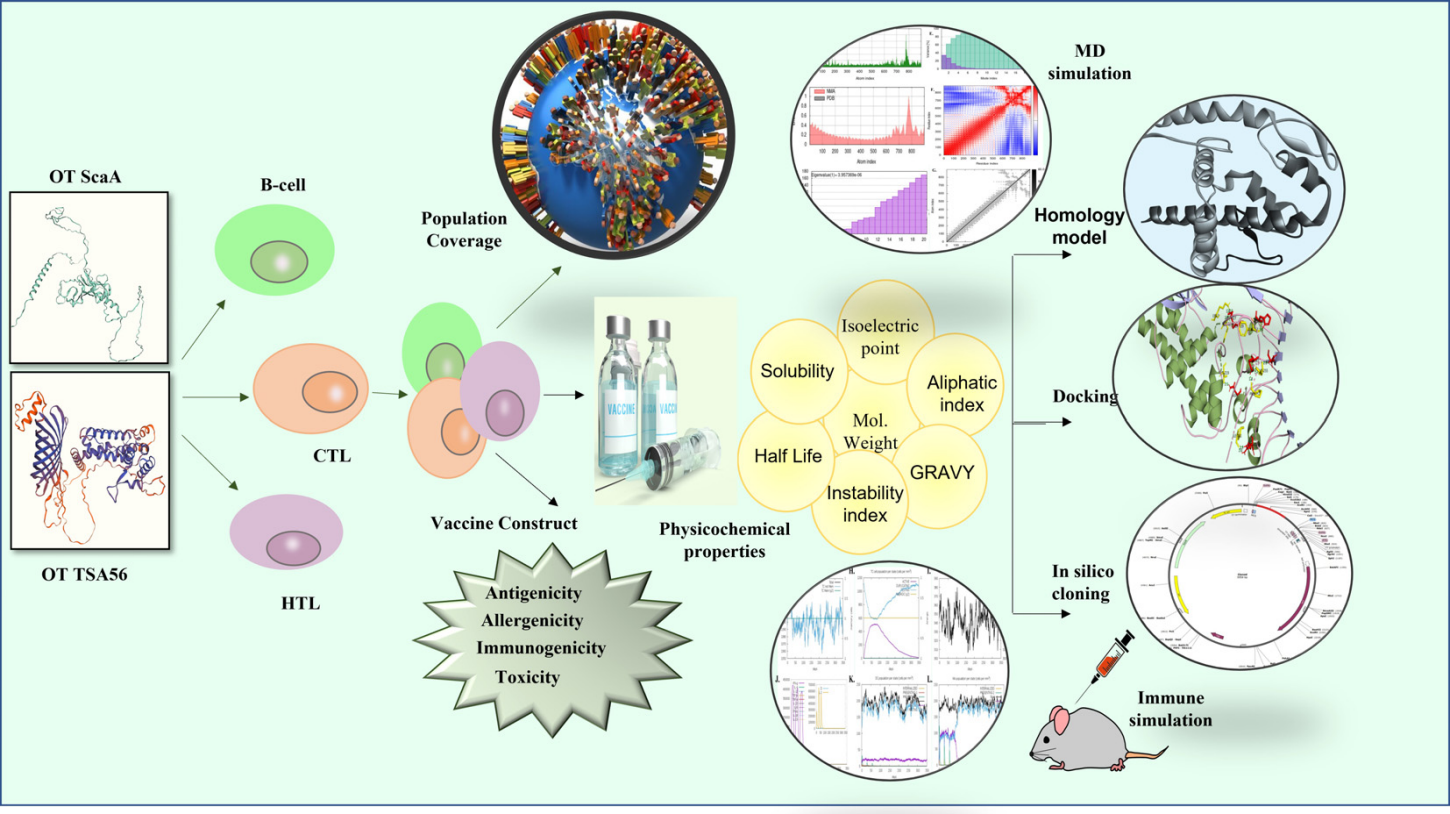
The complete hierarchy of the steps for the multi-epitope vaccine design used in the current study.

Using the C-ImmSim server, in silico immune simulations were carried out to evaluate the immunogenic properties of a multi-epitope vaccine under real-world settings (42). The duration between doses 1 and 2 for vaccination should be at least 4 weeks. As a result, 3 injections containing one thousand vaccine proteins were administered 4 weeks apart at 1, 84, and 168 time-steps (each time-step equals 8 h in real life, and time-step 1 is injection at time = 0), for a total of 1050 simulation steps (parameters were set in the C-ImmSim immune simulator). Three injections of selected peptides were administrated at four-week intervals to simulate repeated antigen exposure and study clonal selection in a typical endemic area. The graph was used to determine the Simpson index (D), a metric of variety. **Figure 1** depicts the entire process used in the reverse vaccinology analysis.

## 3 Results

### 3.1 Retrieval and phylogenetic analysis of target proteins

The complete proteome of the Gilliam strain was retrieved from NCBI to extract virulent TSA56 and ScaA proteins in FASTA format for peptide vaccine designing. The proteins were found to be antigen and non-allergen, with antigenicity scores of 0.79 and 0.82 for TSA56 and ScaA, respectively.

### 3.2 Evaluation of B-cell, CTL and HTL epitopes

Peptide mapping using ABCPred on the FASTA sequences of TSA56 and ScaA proteins revealed a total of 16 epitopes at the default threshold >0.51 and window length of 10. These peptides were selected by antigenicity and non-allergenicity. Among these, two 10mer peptides (TGAESTRLDS and SAEVEVGKGK of TSA56) with the most favorable ABCPred server score were chosen for additional assessment of antigenicity, toxicity, and allergenicity, shown in **Table 1**.

**Table 1:** B cell epitopes prediction for scrub typhus vaccine construct.

Sequence	Start position	Score	Predicted Antigenicity	Score	Predicted Allergenicity	ToxinPred Prediction
SAEVEVGKGK	100	0.78	Antigen	1.7382	Non allergen	Non-toxin
TGAESTRLDS	47	0.57	Antigen	2.0162	Non allergen	Non-toxin

Antigenic determinants presented by MHC-I elicit a cellular immunological response. This kind of reaction often stimulates the production of cytotoxic cells. **Table 2** summarizes the final selection of 6 epitopes for vaccine construction among the top 22 and 7 sequences of epitopes from TSA56 and ScaA protein. To determine HTL epitopes for all structural proteins, the IEDB server for human MHC-II alleles was used. Hence the epitopes that had an SMM align IC50 value less than 50 and a percentile rank of 1 or lower were chosen. Based on the low percentile rank and a strong affinity concerning all the HLA supertypes, the epitopes YSINPLMASVGVRYN and RKRFKLTPPQPTIMP were appointed for consideration in the vaccine design (**Table 3**). All the candidate epitopes that passed the screening process were antigenic, non-allergen, and non-toxic.

**Table 2:** Shortlisted CTL epitopes for the final vaccine construct.

CTL	Peptide	Start	MHC	Affinity	Score	Predicted Allergenicity	Predicted Immunogenicity	Predicted Toxicity
TSA 56	KLQRHAGVK	375	HLA-A*03:01	28.15	1.0316	Non-allergen	0.13553	Non- toxin
	IYAGVGAGL	448	HLA-A*24:02	428.41	0.9248	Non-allergen	0.17172	Non- toxin
	ASVGVRYNF	516	HLA-B*58:01	84.91	1.8822	Non-allergen	0.12952	Non- toxin
ScaA	IFFTTLFTI	10	HLA-A*24:02	78.24	0.8279	Non allergen	0.2259	Non-toxic
	PTVGVRHSY	1378	HLA-A*26:01	1292.11	1.268	Non allergen	0.06706	Non-toxic
	SKFGGGNSL	741	HLA-B*39:01	40.78	1.3881	Non allergen	0.03488	Non-toxic

**Table 3:** Selected HTL epitopes for the final vaccine construct.

Alleles	Start	Peptide	ic50	Score	Predicted Allergenicity	Predicted Toxicity
HLA-DRB1*01:01 HLA-DRB1*13:02 HLA-DRB1*07:01 HLA-DRB1*09:01 HLA-DRB1*12:01 HLA-DRB1*03:01 HLA-DRB5*01:01 HLA-DRB1*15:01 HLA-DQA1*05:01/DQB1*03:01 HLA-DRB3*02:02 HLA-DRB1*11:01 HLA-DQA1*01:02/DQB1*06:02 HLA-DRB4*01:01 HLA-DRB1*08:02 HLA-DRB1*04:05 HLA-DPA1*03:01/DPB1*04:02 HLA-DRB1*04:01 HLA-DPA1*01:03/DPB1*04:01 HLA-DQA1*04:01/DQB1*04:02 HLA-DPA1*02:01/DPB1*01:01 HLA-DPA1*01:03/DPB1*02:01 HLA-DPA1*02:01/DPB1*14:01 HLA-DQA1*05:01/DQB1*02:01	509	YSINPLMASVGVRYN	7.6	1.0399	non-allergen	Non-toxin
HLA-DRB1*01:01 HLA-DRB1*04:05 HLA-DRB3*02:02 HLA-DRB1*07:01 HLA-DRB1*09:01 HLA-DRB5*01:01 HLA-DPA1*02:01/DPB1*14:01 HLA-DRB1*13:02 HLA-DRB1*11:01 HLA-DRB4*01:01 HLA-DRB1*04:01 HLA-DPA1*02:01/DPB1*01:01 HLA-DRB1*08:02 HLA-DQA1*05:01/DQB1*03:01 HLA-DRB3*01:01 HLA-DPA1*03:01/DPB1*04:02 HLA-DPA1*01:03/DPB1*04:01 HLA-DRB1*15:01 HLA-DRB1*03:01 HLA-DRB1*12:01	129	RKRFKLTPPQPTIMP	8.3	1.0248	non-allergen	Non-toxin

### 3.3 Designing of vaccine construct and evaluation

**Figure 2:**
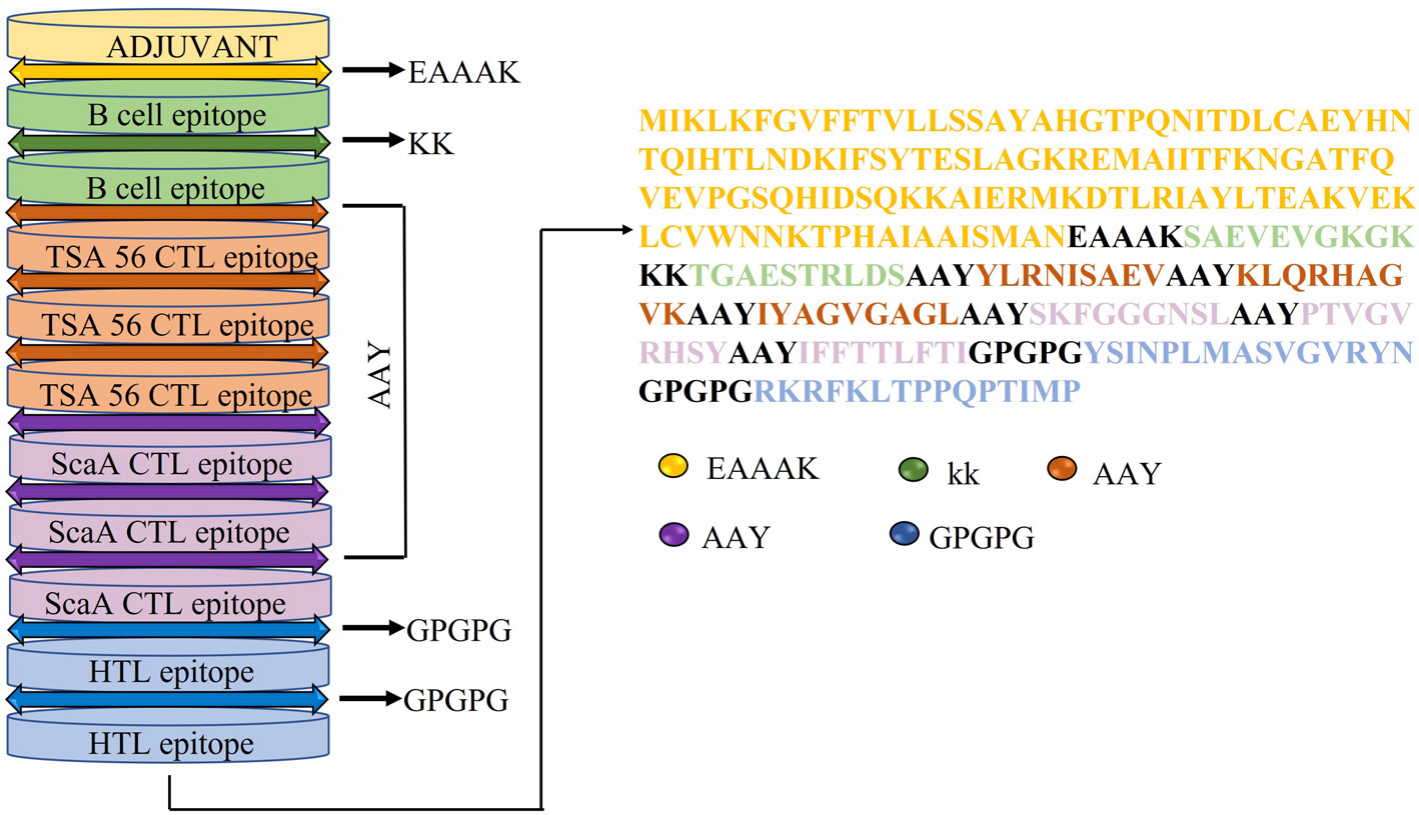
Schematic representation of the final vaccine construct that includes adjuvant, LBL, CTL, and HTL epitopes shown in different colors (top to bottom) linked by EAAAK, KK, AAY, GPGPG linkers respectively.

Six CTLs, two HTLs, and two BCLs were used to create the final chimeric vaccine. EAAAK peptide linker is used to connect the N-terminus of the vaccine model to cholera toxin subunit B (ACO36766.1) adjuvant, and KK, AAY, GPGPG linkers are used to separate each BCL, CTL, and HTL epitopes respectively (**Figure 2**). The physicochemical property of the multi subunit vaccine is depicted in **Supplementary Table 1**.

### 3.4 Prediction and assessment of vaccine homology model

**Figure 3:**
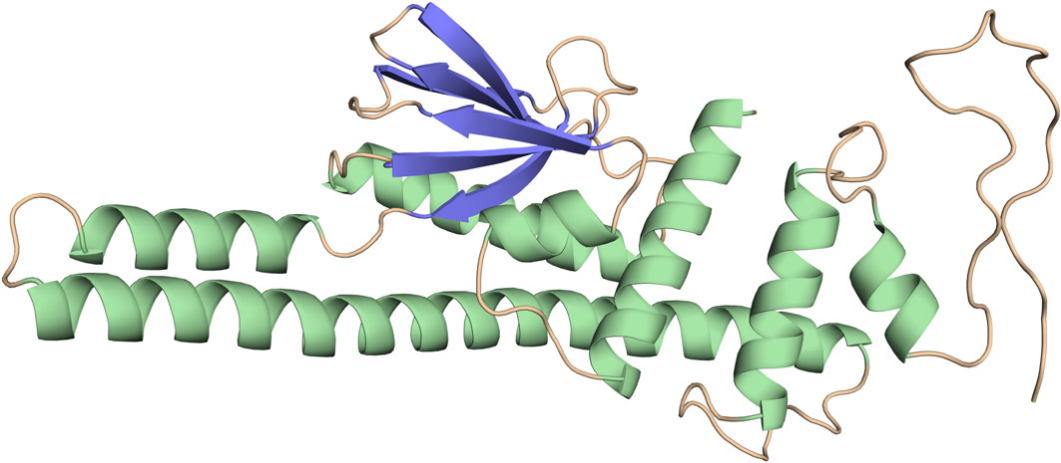
Tertiary structure of the vaccine construct.

PSIPRED was used to make predictions about the secondary structure of the final vaccine construct based on its amino acid sequence (**Supplementary Figure 1**). It was found that the protein structure consists of 44.87% alpha helices, 26.24% random coils, 21.67% extended strands, and 7.22% beta turns. Robetta server created a 3D model of the vaccine construct (**Figure 3**), and model-2 was chosen merely because it had the greatest TMscore. If the TM-score was more than 0.5, signifying the model has been calibrated; the score was from 0 to 1. Using GalaxyRefine2, we were able to refine the projected 3D structure. The following factors were considered while selecting the optimal model for further study; Rama favored 95.5; RMSD, 0.327; GDT-HA, 0.98; Mol probability, 1.75; Clash score, 8.0; Poor rotamer, 0.5. When compared to the initial structure created by the Robetta server, an examination of the Ramachandran Plot of the refined protein acquired by GalaxyRefine indicated improved findings. Similarly, the model obtained via GalaxyRefine exhibits superior stereochemical quality as determined by further structural validation methods like ERRAT (92.43%) and verify 3D (81.73%) shown in **Supplementary Figure 2**.

### 3.5 mRNA structure

As the secondary structure of mRNA plays a crucial role in translation initiation, elongation, and mRNA synthesis, its prediction is of paramount importance. Using the Mfold web server, the free energy associated with the whole mRNA structure was calculated. As shown in **Supplementary Figure 3**, the secondary RNA structure has a minimum free energy of G= 105.27 kcal/mol. This value represents the proteins’ host-based stability and translation efficiency. Enhanced mRNA stability correlates with a higher rate of expression.

### 3.6 Cluster prediction of MHC alleles

MHCcluster 2.0, a web-based program, analyzed clusters of MHC-I and MHC-II alleles that may interact with the projected epitopes. Phylogenetic allele groupings are automatically generated by the program. The results of the experiments are depicted in **Supplementary Figure 4**, where the red areas denote particularly robust interactions and yellow areas, relatively weak interactions.

### 3.8 Discontinuous B-cell epitope analysis

**Figure 4:**
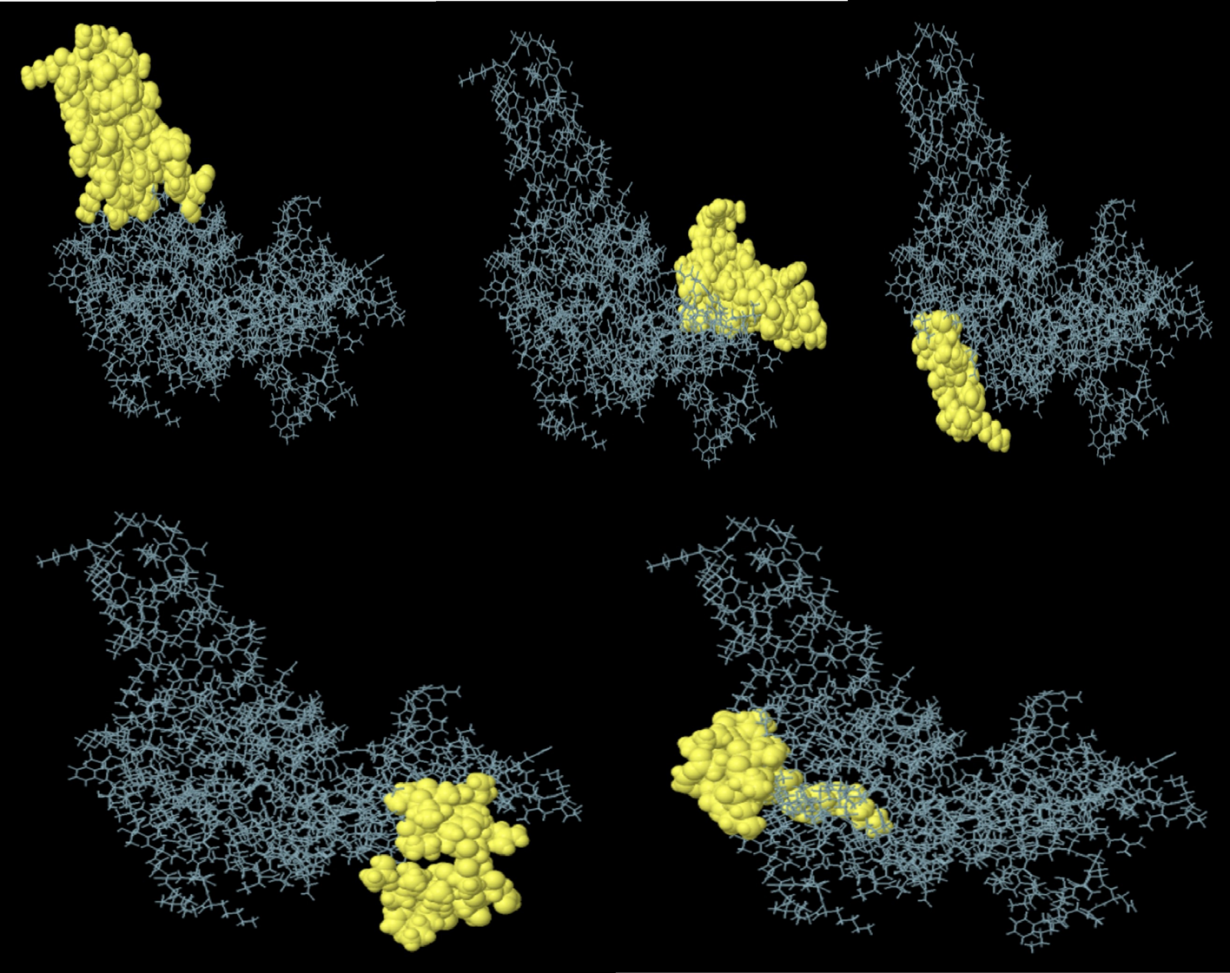
Conformational B-cell epitopes present in the vaccine where the light yellow spheres show the epitopes.

The discontinuous or conformational B-cell epitopes in the engineered vaccines were predicted using the Ellipro tool from the IEDB database with the basic parameters (maximum distance 6 Å and minimum score 0.5). The improved Thornton technique employing the residues clustering algorithm is the basis of these findings. As demonstrated in **Figure 4** and **Table 4**, the prediction is made by taking into account the residual protein index (PI), neighbor residue clustering, and protein shape.

**Table 4:** Predicted conformational B-cell epitopes of the constructed vaccine.

Number	Rresidues	No. of residues	Score
1	N124, A126, A127, A128, K129, S130, A131, E132, V133, E134, V135, G136, K137, G138, K139, K140, K141, T142, G143, A144, E145, S146, T147, R148, L149, D150, S151, A152, A153, Y154, Y155, L156, R157, N158, I159, E162	36	0.787
2	G224, P225, G226, P227, G228, Y229, S230, I231, N232, P233, L234, M235, A236, S237, V238, G239, V240, R241, Y242, N243, G244, P245, G246, P247, G248, R249, K250, F252, K253, L254, T255, P256, P257, Q258, P259, T260, I261, M262, P263	39	0.787
3	Y48, T49, E50, S51, L52, A53, G54, K55, R56, E57, M58, I60, R94	13	0.732
4	A185, A188, A189, S191, K192, F193, G194, G195, G196, N197, S198, L199, A200, A201, Y202, P203, T204, V205, G206, V207, R208	21	0.699
5	D43, K44, F46, S47, T62, K64, N65, G66, A67, T68, Y97, L98, T99, E100, A101, K112, T113, P114	18	0.589

### 3.9 Disulfide bond engineering

**Figure 5:**
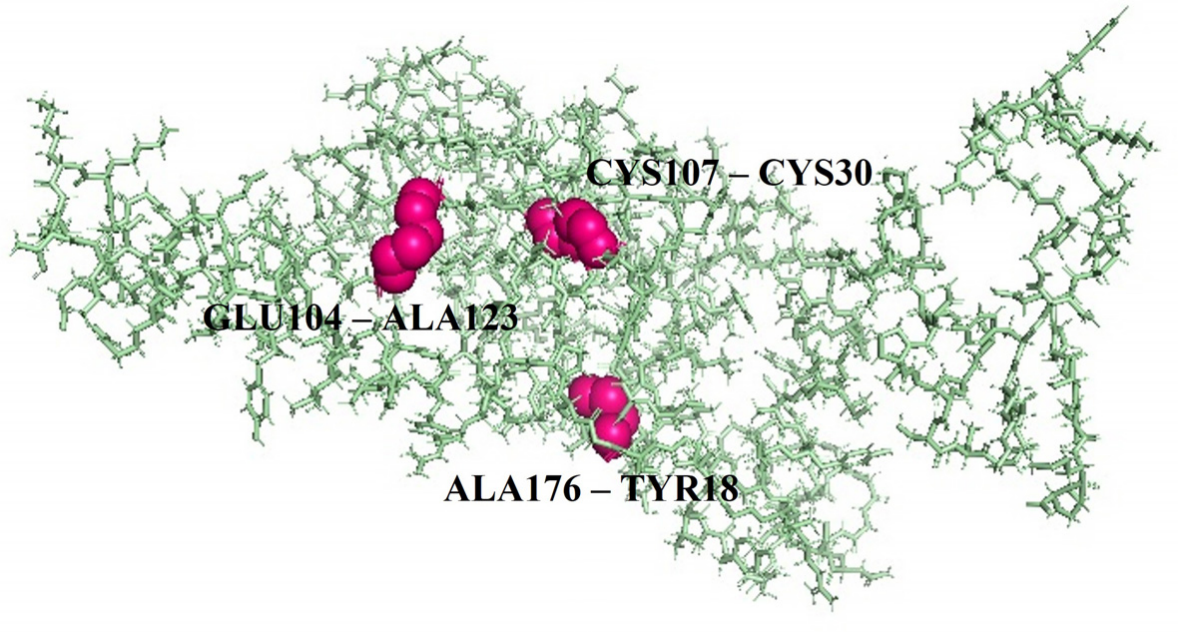
Figure showing the mutant form of vaccine construct by disulfide bond engineering for stability where three pairs of amino acids are represented in pink color.

By using certain geometric conformations, disulfide engineering was used to stabilize the vaccine construct. It was projected that 60 pairs of amino acid residues may create a disulfide bond through the DbD3 server. Two of them TYR18- ALA176, and CYS107- ALA118, which were substituted by cysteine residues, could form disulfide bonds after being assessed by χ3 and Beta-factor energy parameters (**Figure 5**). Based on the chi3 value of -73.37 and – 85, the energy values are 2.45 and 2.41, respectively.

### 3.10 Conservancy analysis and population coverage of vaccine construct

**Figure 6:**
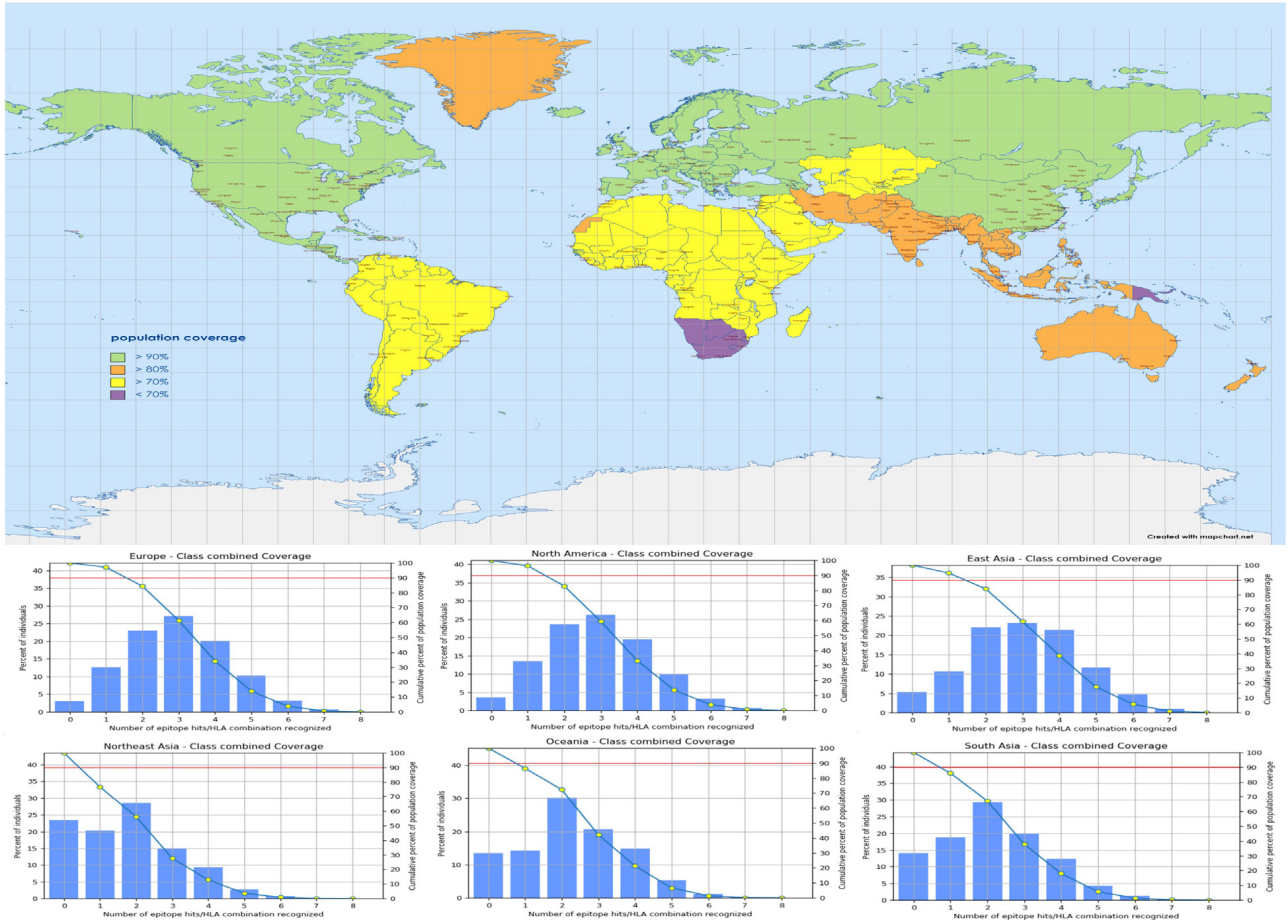
The map represents the worldwide population coverage with combined MHC epitopes based on their respective HLA binding alleles.

To create a universal broad-spectrum vaccination, conserved epitopes against many strains are required. Epitope conservation study about the other OT variants was performed using the IEDB conservation analysis program. All BCL, CTL, and HTL epitopes demonstrated 100% conservation at a sequence identity threshold >60%. Each of the six CD8+ (CTL) and two CD+ (HTL) epitopes was assigned to an allele by the MHC-I and MHC-II prediction server at IEDB. Hence the best binders for the population coverage study were chosen from alleles with IC50 values below 50. In a global examination of MHC-I and MHC-II epitope coverage, an average coverage of 94.35% was found. On the other hand, combining MHC-I and MHC-II epitope coverage was highest in Europe (96.36%), East Asia (94.44%), West Indies (86.52%), Oceania (85.50%), and South Asia (85.50%) shown in **Figure 6**.

### 3.11 Molecular docking

**Figure 7:**
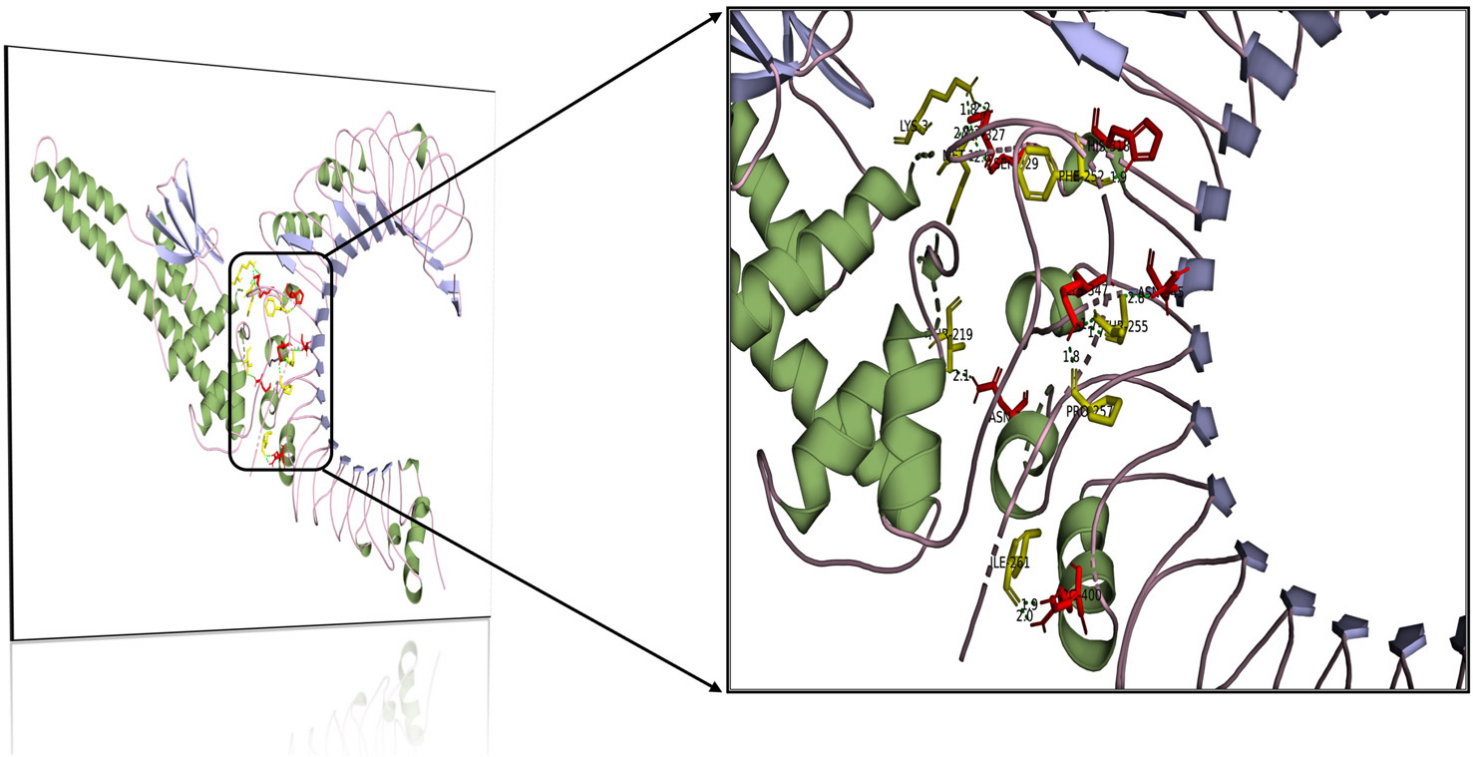
Molecular interaction of multi-epitope vaccine construct docked with TLR-2.

The assimilation of bacterial proteins and their stimulation of interferon and interleukin production in the immune system defense against infections are largely controlled by Toll-like receptors. The ability of the engineered vaccination to bind to the human Toll-like receptor was investigated in the current study. The interaction between the immune cells and the vaccine component is essential for the development of a robust immune response. ClusPro generates 30 unique clusters with increased interaction energies. The first cluster, with the lowest energy value (-1079.2 Kcal/mol) was chosen for further study. **Table 5** lists the nine residues in the vaccine TLR-2 docked complex that form hydrogen bonds with one another (**Figure 7**).

**Table 5:** Results of the molecular docking analysis of the selected epitopes with TLR2 receptor interaction.

TLR-2	Vaccine	Distance
ARG400	ILE261	1.9
ASN376	THR219	2.1
LYS347	PRO257	1.8
LYS347	THR255	1.7
ASN345	THR255	2.8
HIS318	PHE252	1.9
ASP327	LYS3	1.8
ASP327	MET1	2.1
SER329	MET1	2.6

### 3.12 MD simulation of vaccine-receptor docking complex

**Figure 8:**
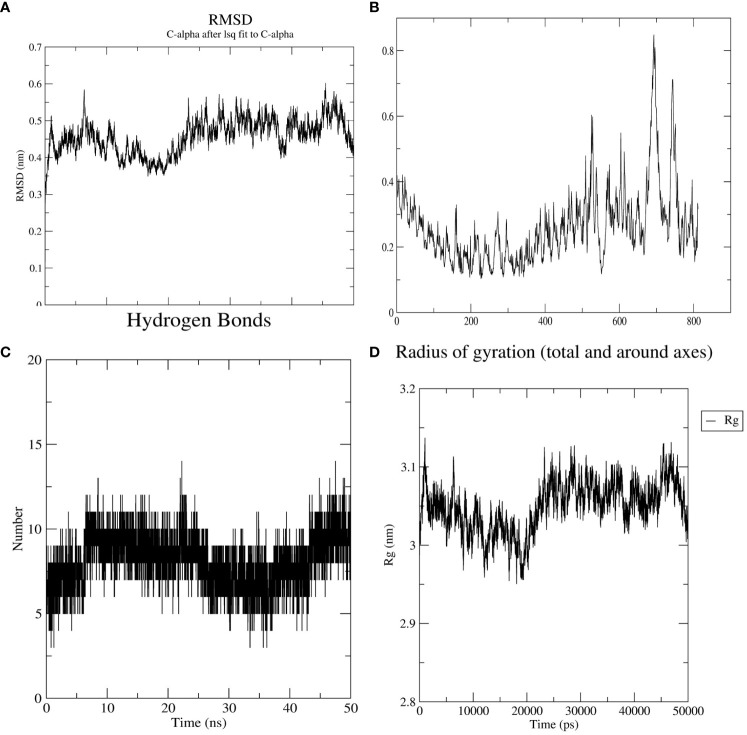
The MD simulation plot represents the interaction and the stability of the atoms and molecules between vaccine construct and TLR2 **(A)** root mean square deviation (RMSD) of the receptor−ligand complex shows infinitesimal deviation and becomes stable at the period of 6 ns, **(B)** Root mean square fluctuation plot (RMSF) representing the fluctuation of side chain residues. **(C, D)** Pressure and temperature plots concerning the time.

As an input for the MD simulation, the best molecular docking complex was chosen. The complex was then subjected to a 50ns MD simulation, with the analysis focusing on key metrics such as root mean square deviation (RMSD), root mean square fluctuation (RMSF), the number of hydrogen bonds (H-bonds), and radius of gyration (Rg). The average RMSD of the receptor-vaccine combination, calculated for all atoms was 0.45 (**Figure 8**). As a result of these favorable interactions, a persistent vaccine-receptor complex may develop. The RMSF describes the residue-by-residue dynamics of a protein about its starting location. The RMSF of the protein atoms was analyzed to determine the conformational behavior of the ligand-receptor complex at the residual level. The average RMSF value was 0.26nm (**Figure 8**). Hydrogen bond interaction plays an essential role in both protein structural stabilization and protein-ligand identification. H-bond formation in the vaccine-receptor complex was also studied using MD simulation to shed light on the possibility of selective intermolecular interactions and the specificity of interactions. On average, nine H-bonds were formed in the vaccine-receptor complex (**Figure 8**). During the simulation, the average radius of gyration (Rg) for the vaccine-TLR2 complex was determined to be 3 nm. The findings demonstrated that the complex’s compactness is enhanced after positive contact between the vaccine protein and the TLR-2 receptor.

### 3.13 Codon optimization and in silico cloning

**Figure 9:**
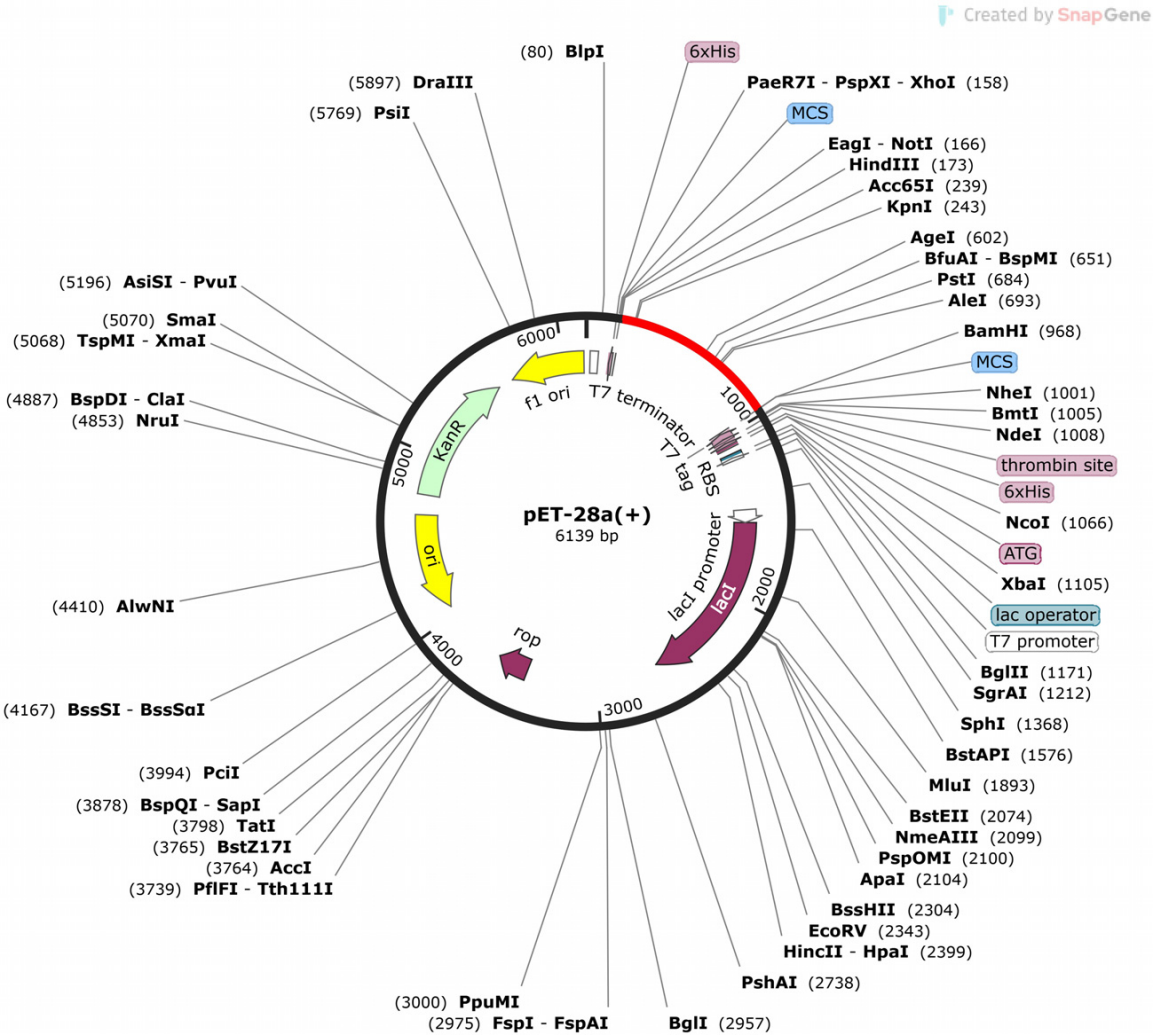
Restriction cloning of final multi-epitope vaccine by using pET28a (+) expression vector in the *in silico* space. The black circle indicates the vector and the red part is the place where the vaccine is inserted.

The serological analysis is the first stage in inspecting a vaccine candidate, and this needs the expression of the vaccine in an appropriate expression system. As an expression system, we settled on *E. coli* optimized cloning, and expression in the *E. coli* K12 strain is made easier with the help of the Java Codon adaptation tool (JCAT). The GC content of the modified sequence was predicted to be 50.19, and the codon adaptation index (CAI) was 0.97. Codon use in the optimal multi-epitope vaccine gene is depicted graphically in **Figure 9**. To execute in silico cloning, restriction enzyme sites were screened in the codon-optimized vaccine construct sequence; HindIII and BamHI were added at the N and C-terminals. After inserting the vaccine construct into pET-28a (+) vector, the functional clone was 6139bp (**Figure 9**).

### 3.14 Immune simulation

**Figure 10:**
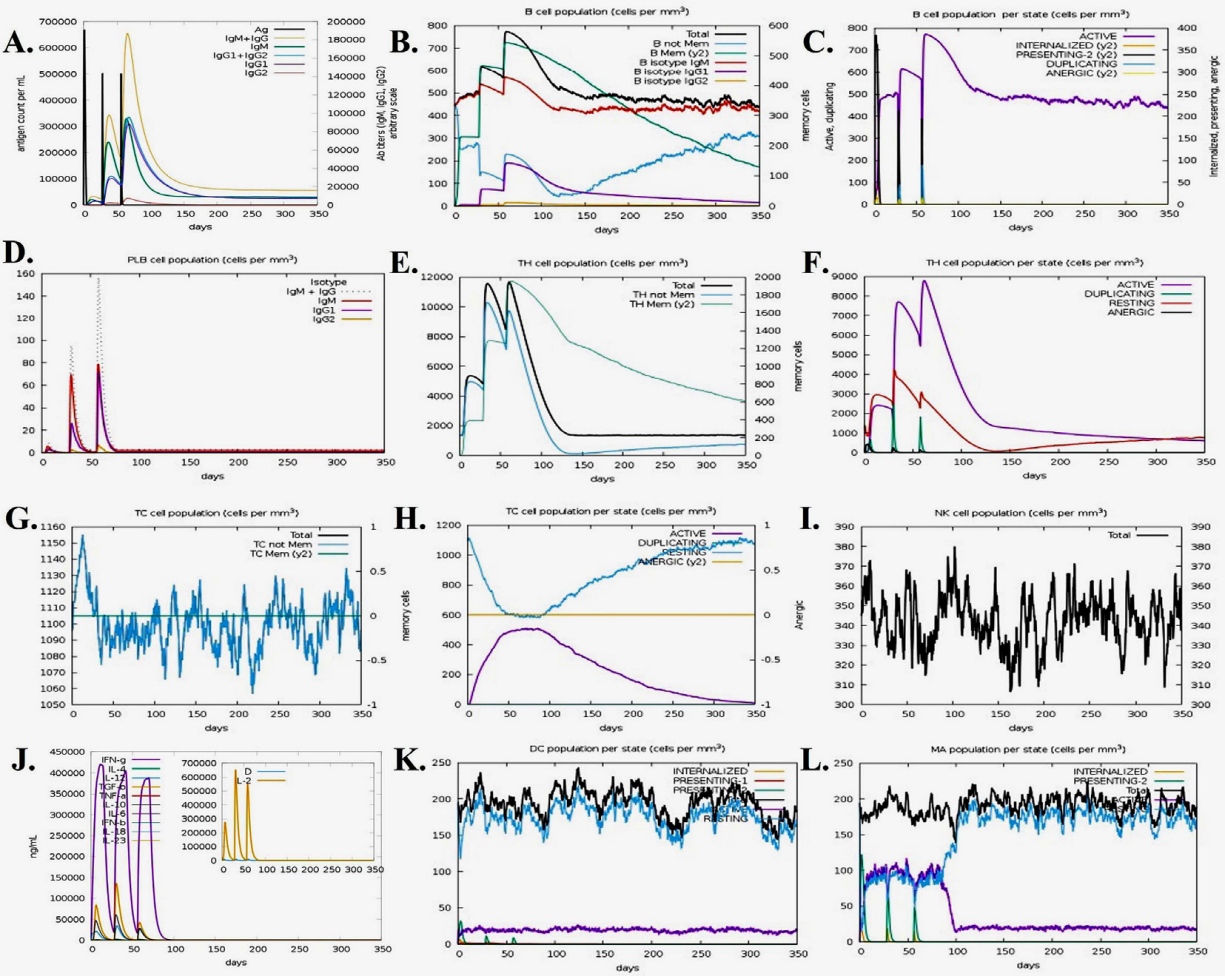
Immune simulation of the predicted vaccine; **(A)** Immunoglobulin and immune complex response to the antigen, **(B)** B lymphocyte population count, **(C)** B lymphocyte population per entity state, **(D)** plasma B lymphocyte count, **(E)** CD4 T helper lymphocyte count, **(F)** CD4 helper lymphocyte count by entity state, **(G)** CD8 T-cytotoxic lymphocyte count, **(H)** CD8 T-cytotoxic lymphocyte count per entity state, **(I)** natural killer cell population, **(J)** different cytokine and interleukin concentration, **(K)** dendritic cells per state and **(L)** macrophage population per state.

The immune simulation for the designed vaccine was conducted using the C-ImmSim server. This server models the immune response generated by key mammalian immune components, including the thymus (T cells), bone marrow (lymph and bone marrow cells), and lymphoid organs. The simulation results provide valuable insights into the potential immunogenicity of the vaccine and the type of immune response it might elicit in humans. After each of the three vaccination injections, the modeling research predicted that the major immune response to the antigenic pieces would expand dramatically, as seen by the gradual increase in concentrations of different immunoglobulins. Once again, it was demonstrated that primary immunological activation boosted subsequent immune responses. The immune simulation study was conducted for the vaccine complexes to explore the generation of adaptive immunity and also the immune interactions. The immune simulation study illustrated that after every injection dose, the primary immune response was increased significantly as gradual elevation or decrease rates of the different immunoglobulins were observed. Moreover, the secondary immune response was also increased (**Figure 10A**). The increasing rate of active B-cells (**Figures 10B, C**), plasma B-cell (**Figure 10D**), helper T-cells (**Figures 10E, F**), and regulatory and cytotoxic T-cells (**Figures 10G–I**) was observed. The vaccine protein was also capable of forming a vast number of different types of cytokines. **Figure 10J** shows the concentration of different cytokines and interleukins. These results indicated after every injection, a strong secondary immune response, increasing clearance of antigens, and strong immune memory generation occur. Moreover, good antigen presentation was also observed by these antigen-presenting cells from dendritic cell and macrophage cell concentrations (**Figures 10K, L**).

## 4 Discussion

Immunotherapy has emerged as a leading method for combating infectious diseases and saving lives. Bioinformatics, vaccinomics, and immunoinformatics are the new methodologies and technologies used in vaccine production that reduce the time and resources required to create a vaccine (43). Potential negative aspects of conventional vaccine production techniques include challenges with adequate culture of the microorganisms and undesirable immune responses arising from improper attenuation. The scientific community has generally warmed up to these software and database techniques (44, 45). As a result of this, research took advantage of the widely used in silico-based methodologies to build possible vaccines against *O. tsutsugamushi* for combating Scrub typhus disease. Antigens of *O. tsutsugamushi* that promote both humoral and T cell-mediated defense would be ideal for vaccination against scrub typhus. Another possibility that can be developed and evaluated is a multiplex subunit vaccine that induces substantial humoral and T cell-mediated protection.

To the greatest extent of our understanding, this is the first paper to detail the *in silico* development of a multi-epitope vaccine against scrub typhus. Antigenic epitopes from two surface-exposed highly pathogenic proteins like TSA56 and ScaA were used to create the vaccine design. According to research by Ha NY et al., ScaA acts as an adhesion factor for bacteria and an anti-ScaA antibody effectively blocks bacterial infection of host cells. When coupled with TSA58, a key outer membrane protein of *O. tsutsugamushi*, immunization with ScaA not only offers considerable protection against heterogeneous strains but additionally confers immunity against fatal challenges with the homologous strain (17). **Supplementary Table 2** Provides the servers used in the analysis.

Another important immune cell is the CD4+ T cell (HTL), which may switch between the Th1 and Th2 phenotypes to elicit different types of immunological responses (46). The Th1 response stimulates the production of CD8+ T cells, natural killer cells, and macrophages. Antibody synthesis and the elimination of external pathogens are hallmarks of the Th2 immune response (47), which is involved in the activation of B cells, the differentiation of B cells, affinity maturation, and antibody production. The vaccine was docked with the TLR-2 receptor to evaluate the significance of the immune response it might produce. Finally, MD simulation was performed on the docked vaccine-receptor complex up to 50ns to verify the stability of the interaction. The expression of the vaccine construct in the *E. coli* K12 host strain was studied by in silico cloning. The results of the immunological simulation showed that a high level of antibacterial cytokines, as well as humoral and innate immune responses, may be triggered by employing this multi-epitope vaccine. It might therefore be a promising vaccine candidate similar to a comparable multi-epitope vaccine against Pseudomonas infection (9). Finally, this multi-epitope construct certainly contributes to the future development of a broad-spectrum peptide vaccine against *O. tsutsugamushi* bacteria. Vaccine research has shifted its focus in recent years to utilize novel platforms such as virus-like particles (VLPs), DNA, and messenger RNA (48, 49). However, with high production costs and limited manufacturing yield, VLPs do not prefer mammalian expression systems for subunit vaccines (50). When it comes to DNA vaccines, however, adenoviral vectors have been the focus of a great deal of research and extensively evaluated (51, 52).

## 5 Conclusion

In conclusion, there has been a lack of successful strategy in developing a vaccine for 80 years. Insufficient knowledge of immunity to *O. tsutsugamushi*, in particular the criteria for vaccine-induced immunity, limited understanding of immunological memory in scrub typhus, and a failure to address the issue of cross-protection between strains, all contributed to the failure of previous efforts. In sum, our results backed up the ability of the construct to govern promising immune responses against this infectious disease, and this next-generation strategy offered a fresh perspective on creating a highly immunogenic vaccine for scrub typhus.

## Data Availability

The original contributions presented in the study are included in the article/**Supplementary Material**, further inquiries can be directed to the corresponding author/s.

## Supplementary material

Supplementary Figure 1 Prediction of the secondary structure of the multi-epitope vaccine construct.

Supplementary Figure 2
**(A)** Ramachandran plot for the validation of the designed structure **(B, C)** Quality assessment by ERRAT and Verify 3D.

Supplementary Figure 3 Prediction of RNA secondary structure of vaccine construct gene by Mfold.

Supplementary Figure 4 MHC cluster analysis tree map and heat map of both MHC-I and MHC-II epitopes.
