# Supplementary figures and images for: Designing a potent multivalent epitope vaccine candidate against Orientia tsutsugamushi via reverse vaccinology technique - bioinformatics and immunoinformatic approach
Source: Front Immunol. 2025 Feb 13;16:1513245. doi: 10.3389/fimmu.2025.1513245 (PMC11865050; doi:10.3389/fimmu.2025.1513245)

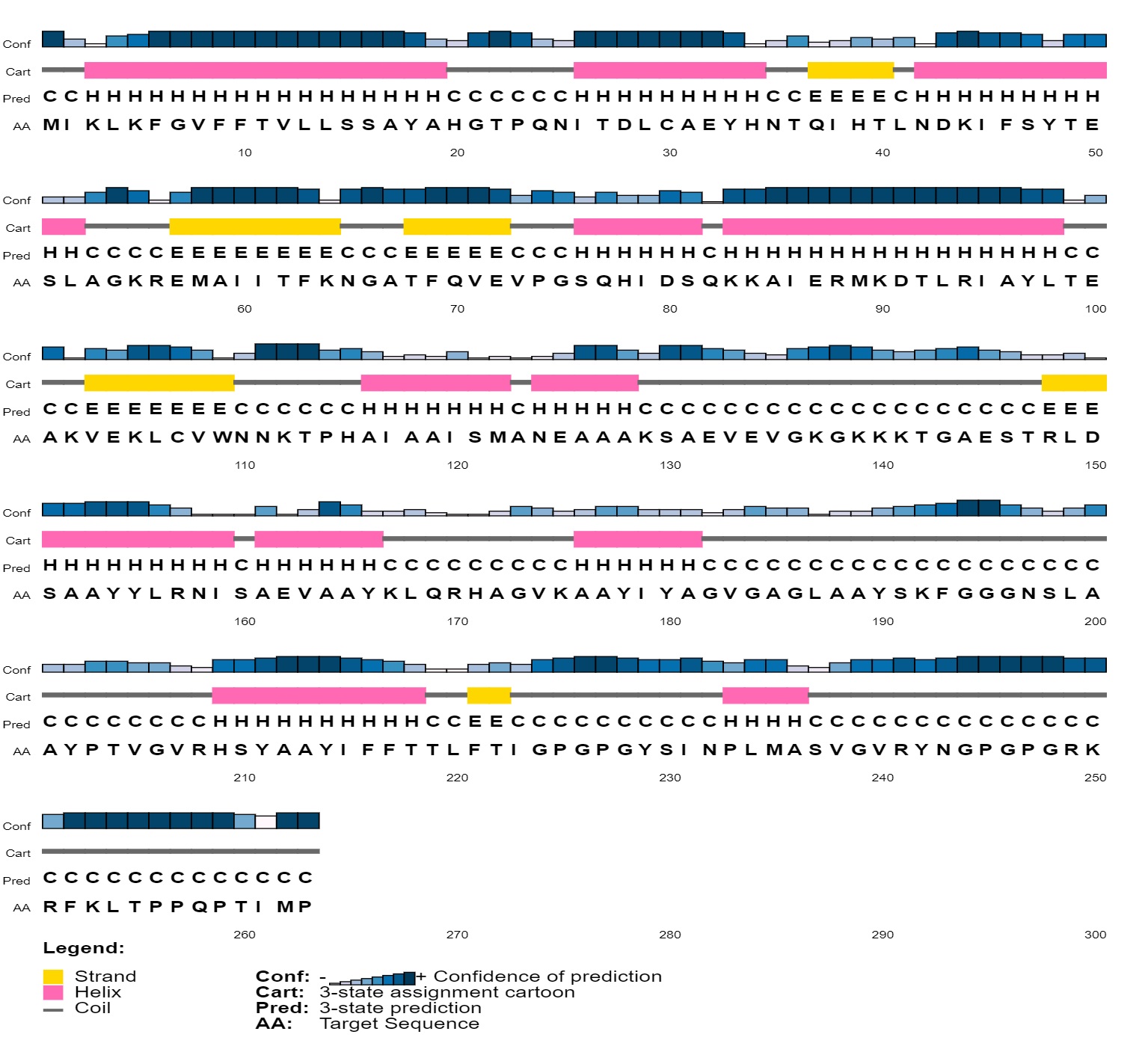

Supplement: Supplementary Figure 1 — Prediction of the secondary structure of the multi-epitope vaccine construct. [file Image1.jpeg]

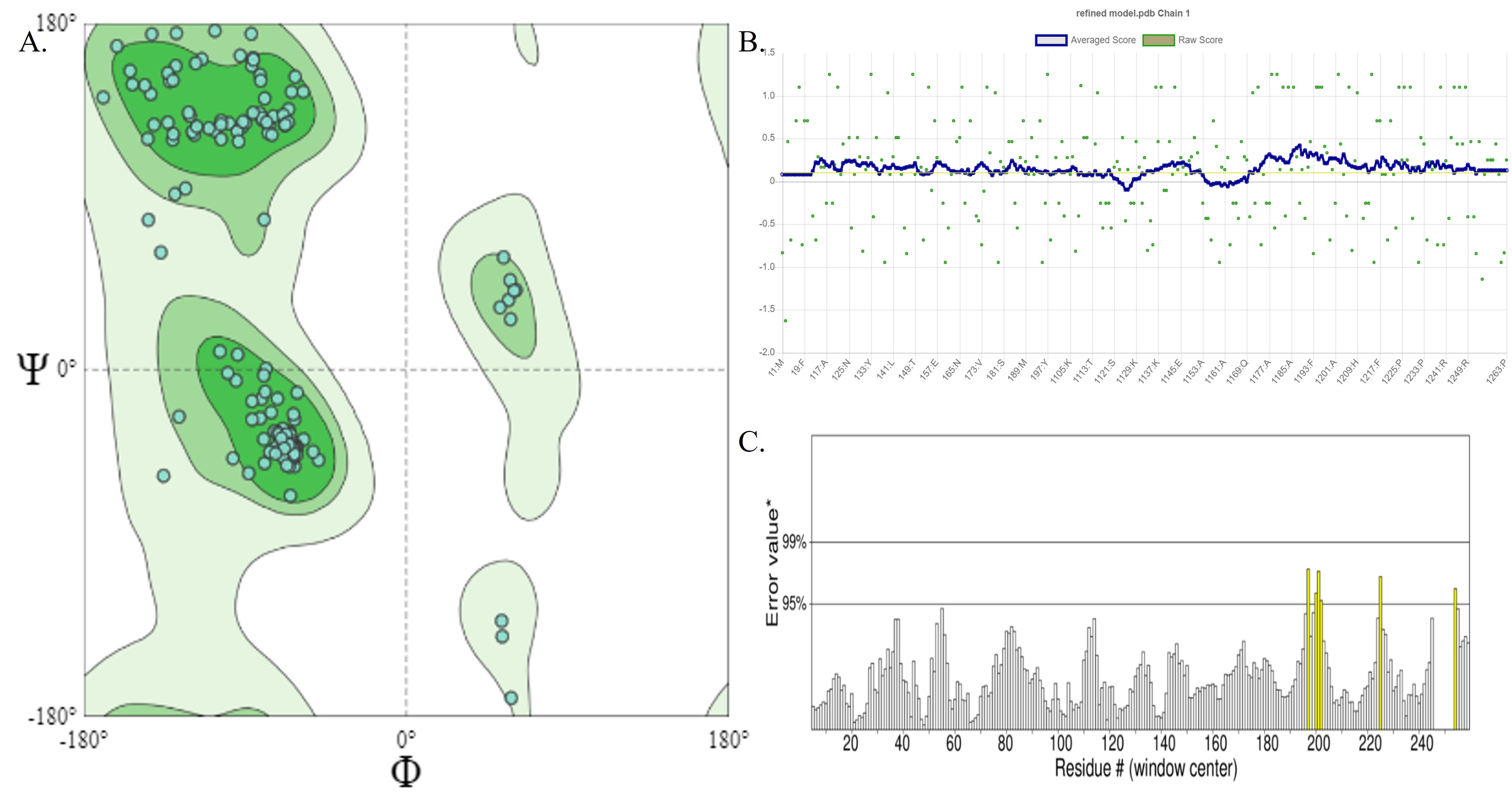

Supplement: Supplementary Figure 2 — (A) Ramachandran plot for the validation of the designed structure (B, C) Quality assessment by ERRAT and Verify 3D. [file Image2.jpeg]

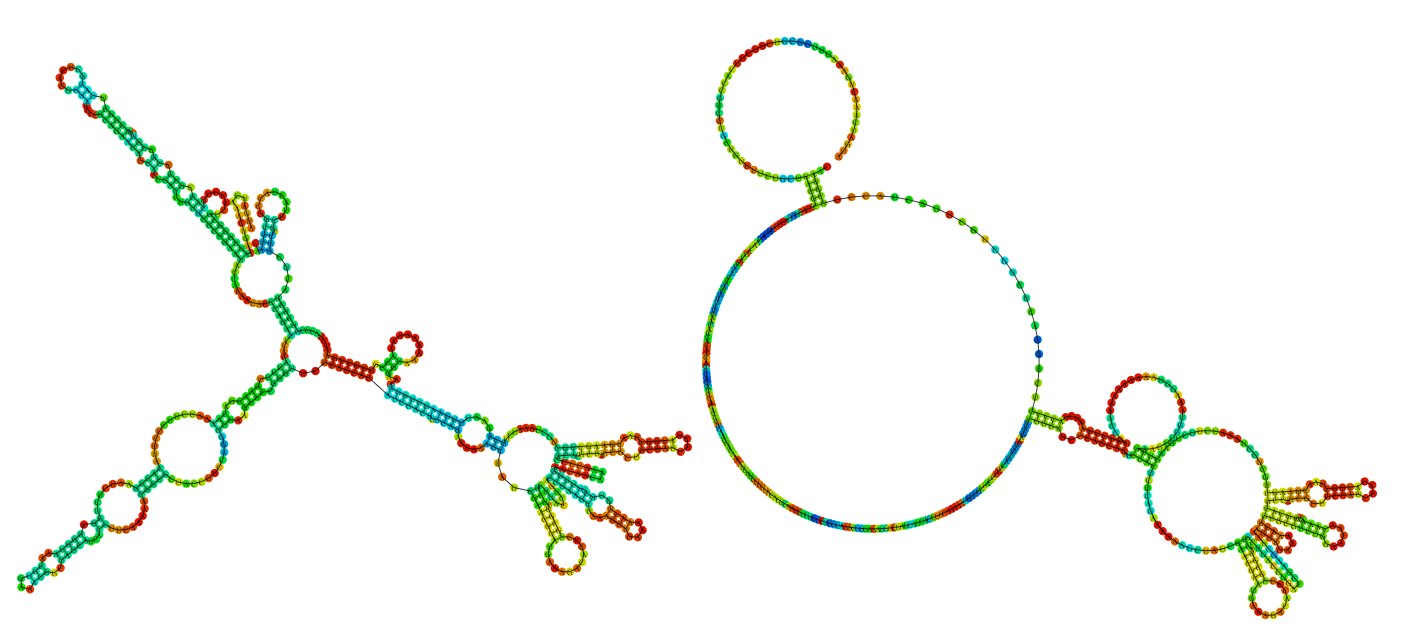

Supplement: Supplementary Figure 3 — Prediction of RNA secondary structure of vaccine construct gene by Mfold. [file Image3.png]

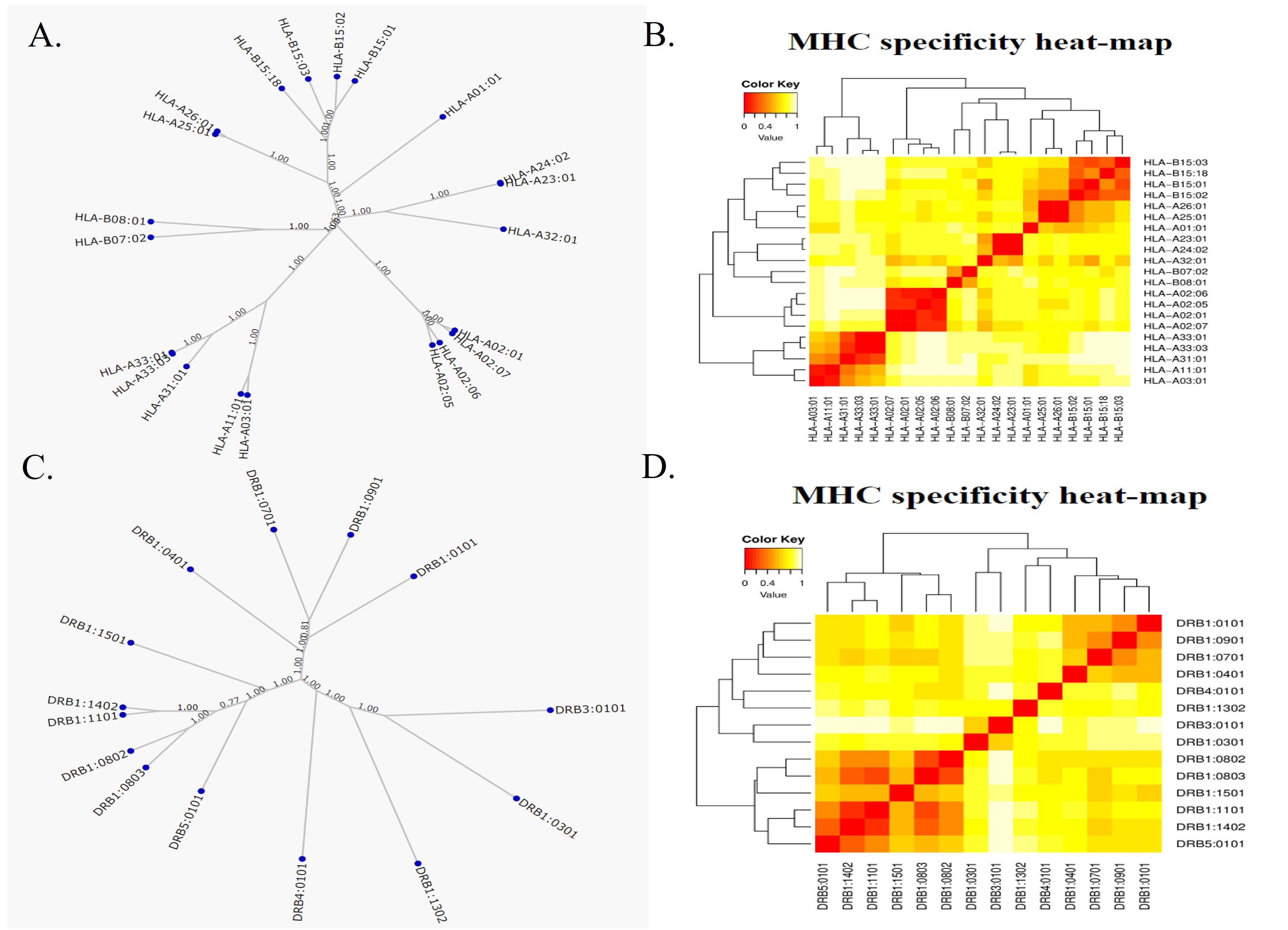

Supplement: Supplementary Figure 4 — MHC cluster analysis tree map and heat map of both MHC-I and MHC-II epitopes. [file Image4.jpeg]
