# Supplementary material for: Designing a potent multivalent epitope vaccine candidate against Orientia tsutsugamushi via reverse vaccinology technique - bioinformatics and immunoinformatic approach
Source: Front Immunol. 2025 Feb 13;16:1513245. doi: 10.3389/fimmu.2025.1513245 (PMC11865050; doi:10.3389/fimmu.2025.1513245)
Supplement: Supplementary file 5 [file Table1.docx]

**Supplementary Table 1:** Physicochemical properties of the vaccine construct

| **Physicochemical Properties** | **Results** |
| --- | --- |
| Number of amino acids | 263 |
| Molecular weight | 28465.77 |
| Theoretical PI | 9.66 |
| Total no of -vely charged residues | 17 |
| Total no of +vely charged residues | 31 |
| Chemical formula | C_1287_H_2019_N_347_O_368_S_8_ |
| Total no of atoms | 4027 |
| Instability index | 32.20 |
| Aliphatic index | 80.27 |
| GRAVY | -0.129 |
| Solubility upon over expression | -0.50 |

**Supplementary Table 2:** Provides the servers used in the analysis.

| **Methods** | **Server used** | **URL** |
| --- | --- | --- |
| BCL epitope prediction | ABCpred | <http://crdd.osdd.net/raghava/abcpred/> |
| CTL epitope prediction | NetMHCpan 4.1 | <https://services.healthtech.dtu.dk/services/NetMHCpan-4.1/> |
| HTL epitope prediction | IEDB | <http://tools.iedb.org/mhcii/> |
| Antigenicity | VaxiJen | <http://www.ddg-pharmfac.net/vaxijen/VaxiJen/VaxiJen.html> |
| Allergenicity | AllerTop | <https://www.ddg-pharmfac.net/AllerTOP/> |
| Toxicity | ToxinPred | <http://crdd.osdd.net/raghava/toxinpred/> |
| Solubility | Scatch | <http://scratch.proteomics.ics.uci.edu/> |
| Physicochemical properties | ExPasy’sProtparam | <https://web.expasy.org/protparam/> |
| mRNA | mFold | <http://www.unafold.org/mfold/applications/dna-folding-form.php> |
| Cluster analysis | MHCcluster 2.0 | <https://services.healthtech.dtu.dk/services/MHCcluster-2.0/> |
| Conservancy analysis | IEDB Conservancy | <http://tools.iedb.org/conservancy/> |
| Population coverage | IEDB Population | <http://tools.iedb.org/population/> |
| Secondary structure | PSIPred | <http://bioinf.cs.ucl.ac.uk/psipred/> |
| Homology modelling | Robetta | <https://robetta.bakerlab.org/> |
| Structure refined | GalaxyRefine2 | <http://galaxy.seoklab.org/refine2> |
| Ramachandran Plot | PROCHECK | <https://www.ebi.ac.uk/thornton-srv/software/PROCHECK/> |
| Quality assessment | SAVES | <https://saves.mbi.ucla.edu/> |
| Linear B-cell epitope | ElliPro | <http://tools.iedb.org/ellipro/> |
| Disulfide bond engineer | DbD2 | <http://cptweb.cpt.wayne.edu/DbD2/index.php> |
| Protein-protein docking | ClusPro | <https://cluspro.bu.edu/login.php> |
| Codon adaptation | jCAT | <http://www.jcat.de/Literature.jsp> |
| Immune Simulation | C-IMMSIM | <https://kraken.iac.rm.cnr.it/C-IMMSIM/> |
